# Altered Spontaneous Brain Activity of Children with Unilateral Amblyopia: A Resting State fMRI Study

**DOI:** 10.1155/2019/3681430

**Published:** 2019-07-25

**Authors:** Peishan Dai, Jinlong Zhang, Jing Wu, Zailiang Chen, Beiji Zou, Ying Wu, Xin Wei, Manyi Xiao

**Affiliations:** ^1^School of Computer Science and Engineering, Central South University, Changsha, Hunan 410083, China; ^2^Hunan Engineering Research Center of Machine Vision and Intelligent Medicine, Central South University, Changsha 410083, China; ^3^Department of Ophthalmology, Second Xiangya Hospital, Central South University, Changsha, Hunan 410083, China; ^4^Hunan Clinical Research Center of Ophthalmic Disease, Changsha, Hunan 410083, China

## Abstract

**Objective:**

This study is aimed at investigating differences in local brain activity and functional connectivity (FC) between children with unilateral amblyopia and healthy controls (HCs) by using resting state functional magnetic resonance imaging (rs-fMRI).

**Methods:**

Local activity and FC analysis methods were used to explore the altered spontaneous brain activity of children with unilateral amblyopia. Local brain function analysis methods included the amplitude of low-frequency fluctuation (ALFF). FC analysis methods consisted of the FC between the primary visual cortex (PVC-FC) and other brain regions and the FC network between regions of interest (ROIs-FC) selected by independent component analysis.

**Results:**

The ALFF in the bilateral frontal, temporal, and occipital lobes in the amblyopia group was lower than that in the HCs. The weakened PVC-FC was mainly concentrated in the frontal lobe and the angular gyrus. The ROIs-FC between the default mode network, salience network, and primary visual cortex network (PVCN) were significantly reduced, whereas the ROIs-FC between the PVCN and the high-level visual cortex network were significantly increased in amblyopia.

**Conclusions:**

Unilateral amblyopia may reduce local brain activity and FC in the dorsal and ventral visual pathways and affect the top-down attentional control. Amblyopia may also alter FC between brain functional networks. These findings may help understand the pathological mechanisms of children with amblyopia.

## 1. Introduction

Amblyopia is a neurodevelopmental disorder of the visual cortex characterized by visual deficiency in an eye that is otherwise physically normal or by a deficiency that is out of proportion with the structural abnormalities of the eyes [1–3], thereby affecting 2%–4% of the general population [4]. Amblyopia is believed to be caused by an abnormal visual experience during the critical visual development period in childhood [5]. Amblyopia is generally correlated with an abnormal ocular alignment (strabismus) or an unequal refractive error between the two eyes (anisometropia) in early life [6]. The peak of brain plasticity is in early childhood [7], so the brain functional mechanism of amblyopia in children should be investigated to administer treatments timely and accurately.

Functional magnetic resonance imaging (fMRI) can be applied to investigate brain activity noninvasively; as such, fMRI is widely used to reveal neuropathological mechanisms in amblyopia [8, 9]. Amblyopia is considered a visual cortex but not a retinal dysfunction [10]. For this reason, brain areas on the vision pathway have been widely explored. Task-related fMRI, which is obtained by stimulating with a visual task, has been used to investigate local brain activities. The lateral geniculate nucleus (LGN) is a relay center in the visual pathway. Miki et al. [11] used task-related fMRI and revealed that LGN activation is diminished during monocular viewing by an eye with anisometropic amblyopia. Hess et al. [12] found functional deficits in the LGN by employing visual stimulus fMRI in human adults. The primary visual cortex (PVC) (V1) has also been widely explored. Goodyear et al. [13] selected a region of interest (ROI) around the V1 and confirmed that the activation region of the amblyopic eye is smaller than that of the normal eye with the same stimulus. Barnes et al. [14] reported a reduced fMRI activation in the V1 and V2 regions. These findings confirmed that the visual impairment of amblyopia may be related to the functional changes in neurons in the V1 region [15–18]. Further research has indicated that the abnormal brain function of amblyopia is not limited to the PVC. Muckli et al. [19] reported that responses of amblyopic eye were progressively reduced on the central visual pathway (V3a/VP, V4/V8, lateral occipital complex (LOC)) compared with the low-level visual areas (V1/V2) when grating stimuli were presented, suggesting that the vision pathway from the PVC to the high-level visual areas of the amblyopic eye may have been impaired. Spiegel et al. [3] used checkerboard stimulation and found a reduced fMRI activation in the V2 and V3 in amblyopia. Simmers et al. [20–22] employed psychophysical methods and revealed that the ventral and dorsal extrastriate functions are affected by amblyopia. These studies have suggested that amblyopia affects the visual pathway, including primary and high-level visual areas.

rs-fMRI can investigate the spontaneous neuronal activity of the human brain [23, 24]. In comparison with task-related fMRI, rs-fMRI is easily performed, is simple in design, and can be easily obtained for most children [9, 25]. Several scholars investigated the spontaneous brain activity and FC of amblyopia through rs-fMRI. Tang et al. [9] and Liang et al. [26] revealed that the amplitude of low-frequency fluctuation (ALFF) of spontaneous brain activity changes in the anisometropic amblyopia group. Lin et al. [1] observed that regional homogeneity (ReHo) value changes in individuals with anisometropic amblyopia. Wang et al. [27, 28] and Ding et al. [29] found decreased FC in the visual pathway in amblyopia. These studies have revealed functional changes in the visual pathway from the ALFF, ReHo, and FC analysis in amblyopia. In addition, they also found functional changes in nonvisual pathway areas. But there were few commonalities between the results. The reasons may be as follows: first, sample characteristics are different. Second, rs-fMRI may reflect spontaneous brain activity, and the noise may be greater than task-related fMRI. Lastly, the effect of amblyopia may not be limited to visual pathways because of neuroplasticity.

In previous rs-fMRI studies, the amblyopia group usually includes a mixture of left eye, right eye, and bilateral amblyopia. But in this study, we chose unilateral amblyopia as a research object to reduce the sample interference between left, right, and bilateral amblyopia. Our hypothesis was that unilateral amblyopia might alter local brain activity and FC, and such an alteration might not completely focus on the visual pathway. To confirm this hypothesis, we analyzed spontaneous brain activity from multiple perspectives, including ALFF, FC in the primary visual cortex (PVC-FC), and FC network between regions of interest (ROIs-FC) analysis.

## 2. Materials and Methods

### 2.1. Participants

The inclusion criteria for the amblyopia group were as follows: the best-corrected visual acuity of 0.3 logMAR (3 years), 0.2 logMAR (4–7 years), 0.1 logMAR (more than 7 years), or two-line (0.2 logMAR) interocular optotype acuity differences without pathology and history of treatment. The inclusion criteria for HCs were as follows: without amblyopia-related diseases and history of treatment. There was no significant difference in age and gender to the amblyopia group.

This study was approved by the Ethics Committee of the Second Xiangya Hospital, Central South University, and in accordance with the Declaration of Helsinki. A written informed consent was obtained from all participants enrolled in the study or from their legal guardians. All participants received detailed eye examinations that included assessments of visual acuity, intraocular pressure and refraction, slit lamp examination, ophthalmoscopy, binocular alignment, ocular motility, and random dot butterfly stereograms.

The participants included 17 individuals with normal vision and 17 individuals with amblyopia, and they were enrolled as two groups of subjects in the study. From each group, 4 individuals whose data contained consistent outliers likely due to excessive head motion were excluded from the analysis. As a result, 13 individuals were retained in the amblyopia group, and the same number of age-matched normally sighted individuals remained in the control group. The individuals in the amblyopia group had left eye amblyopia. Those in the control group had normal or corrected to normal visual acuity in both eyes and reported no history of visual disorders. The demographic information of the participants is summarized in Table 1.

### 2.2. Magnetic Resonance Imaging Data Acquisition

Scans were conducted using a Philips Achieva 3-T magnetic resonance scanner at Trinity MRI, with a Philips 8 Channel head coil. The scanning session included a T1-weighted anatomical scan (TE, 2.7 ms; TR, 5.8 ms; flip angle, 8; voxel size, 1 mm^3^) followed by a blood oxygenation level-dependent fMRI (BOLD-fMRI) scan (single-shot spin-echo echo-planar imaging, parallel imaging [36 slices]). BOLD-fMRI scans were measured with transverse orientation; AP fold-over direction; TE, 2000 ms; TR, 30 s; flip angle, 90°; isotropic 3.5 mm × 3.5 mm × 3.5 mm resolution; FOV, 240 mm × 240 mm × 144 mm; acquisition matrix *M* × *P*, 64 × 64; REC voxel MPS, 1.67 mm × 1.67 mm × 4.0 mm; and time points = 189.

### 2.3. Data Processing

Preprocessing steps were generated using the Data Processing and Analysis for Brain Imaging (DPABI) software package [30]. The steps included DICOM-to-NIFTI transformation, removal of 10 time points, slice timing correction, head motion correction, nuisance covariate regression (six head motion signals, white matter, averaged cerebrospinal flow, and global), standard space normalization (based on the Montreal Neurological Institute coordinate system), smoothing with a 4 mm full width at a half maximum of Gaussian kernel to decrease the spatial noise, and band-pass filtering (0.01 Hz < *f* < 0.08 Hz) of the waveform of each voxel to reduce the effects of low-frequency drift and high-frequency noise [31]. Head motion parameters had no significant difference between the two groups.

### 2.4. ALFF Analysis

After preprocessing, ALFF was computed on each individual's data. The ALFF of the rs-fMRI signal has been widely used to measure the intensity of regional spontaneous brain activity [24, 32–36]. ALFF analysis does not depend on prior knowledge and model, thus avoiding the possible errors in the model and hypothesis dependency. ALFF analysis is conducted to measure the magnitude of the energy from the BOLD signal intensity and indirectly measure the relative activity of the local brain area in the resting state. Such a spontaneous activity in the brain area is generally due to the rhythmic activity of the brain region functionally associated with other brain regions. The brain areas with a high ALFF may indicate an increased spontaneous neuronal activity, whereas the brain areas with a low ALFF may correspond to a decreased spontaneous neuronal activity.

### 2.5. PVC-FC Analysis

#### 2.5.1. ROI Identification

The ROIs of the PVC were defined as the intersections of the gray matter cortex and 17 bilateral Brodmann's areas according to the WFU-atlas [37, 38]. Therefore, two ROIs represent the PVC on the left and right hemispheres.

#### 2.5.2. FC Analysis

The PVC-FC analyis was performed with DPABI. The regional rs-fMRI time series was computed for each ROI by averaging all the voxels within each region at each time point in the time series, resulting in 189 time points for each ROI. The correlation coefficient between the average time series of each ROI and other voxels of the brain was calculated as the connection strength. A two-sample *t*-test was conducted to compare the differences in FC between the amblyopia group and the healthy controls (HCs) at *P* ≤ 0.05 by using GRF correction with a cluster size of >85.

### 2.6. FC Network among ROIs

#### 2.6.1. ROI Selection

Independent component analysis (ICA) was conducted on each participant's smoothened, normalized images by using GIFT v3.0a (Group ICA of fMRI Toolbox) [39]. This analysis was limited to 25 output-only components of the group. From these components, networks of interest (salience network (SN), default mode network (DMN), primary visual cortex network (PVCN), high visual cortex network (HVCN), cerebellum network (CEN), right frontoparietal network (rFPN), and left frontoparietal network (lFPN)) were selected. A nonlinear template matching was conducted to select the “best fit” or the most suitable network component. The template matching steps were as follows: for each component, the average *z*-score of the voxels of the component falling within and outside the template was calculated. Then, the component from the 25 components with the maximum difference in the average *z*-score of voxels falling within and outside the template was selected as the network component that most closely matched the template. The *z*-scores here reflected the degree to which the time series of a given voxel was correlated with the time series corresponding to the specific ICA component. A combined group analysis was performed using the individual best fit network components for the three networks. Individual *t*-statistic images from both groups were used to determine the combined group-level statistical maps by using a single-sample *t*-test. Significant clusters were identified using a voxel-wise statistical height (*P* ≤ 0.01) and extent (*P* ≤ 0.01) thresholds corrected at the whole-brain level.

According to previous results on resting state networks [40], the classical resting state networks were selected from the ICA components. The seed of ROIs of each network was defined on the basis of the peaks of *z*-scores of the ICA clusters and selected (Table 2). The final ROIs were drawn as spheres with a radius of 8 mm centered on the given nodes. The ROIs were created correspondingly on both hemispheres. These ROI selection procedures are widely used in functional and effective connectivity studies [41–44].

#### 2.6.2. FC Analysis

The regionally averaged rs-fMRI time series of each ROI was contracted to calculate the connections between the ROIs, and the correlation between the time series was computed by DPARSF software. Fisher's *r* to *z* transformation was applied to ensure a normal distribution. A single-sample *t*-test was conducted to analyze the FC at a group level, and a two-sample *t*-test was performed to examine the between-group differences (*P* ≤ 0.01, FDR correction, and cluster size > 85).

## 3. Results

### 3.1. Comparison of ALFF in Amblyopia versus HCs

The ALFF values were computed at a voxel level to assess the difference in the intensity of the local brain activity between the two groups.

**Figure 1:**
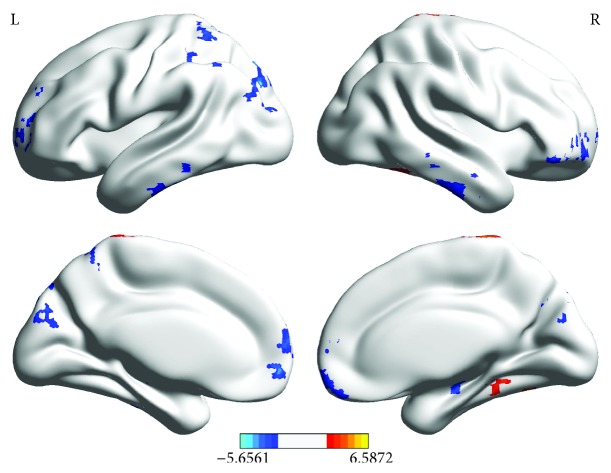
Differences in ALFF between the amblyopia group and HCs (*P* ≤ 0.05, alphasim corrected, and cluster size > 85). The red region indicates that the ALFF of the amblyopia group is significantly higher than that of HCs. The blue region implies that the ALFF of the amblyopia group is significantly lower than that of HCs.

The differences in ALFF between the amblyopia group and HCs are illustrated in the 3D gray cortical model in Figure 1. The differences in ALFF values between both groups as revealed by using the two-sample *t*-test are presented in Table 3.

ALFF analysis showed that the brain regions with lower ALFF values in the amblyopia group than those in the HCs were distributed in the bilateral brain, including frontal, temporal, and occipital lobes. The middle temporal gyrus is a high-level functional area in the visual dorsal information processing stream, and the low ALFF may be related to the attention deficit of amblyopia. The brain regions with higher ALFF values in the amblyopia group than those in HCs were distributed in the right fusiform gyrus, the right caudate nucleus, and the right superior parietal gyrus.

### 3.2. Comparison of PVC-FC in Amblyopia versus HCs

**Figure 2:**
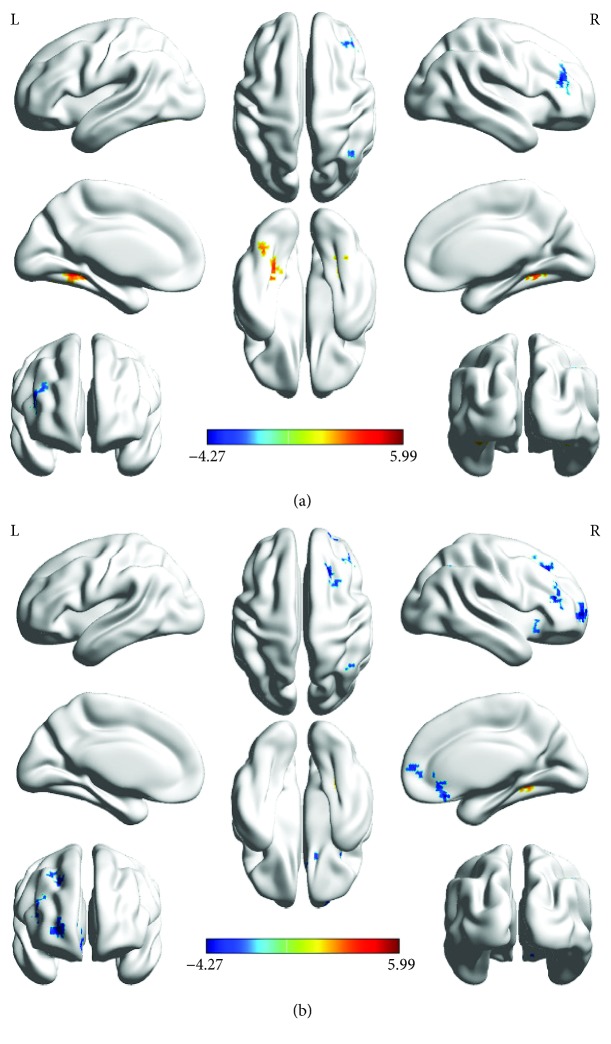
FC between the PVC and other brain regions (*P* ≤ 0.05, GRF corrected, and cluster size > 85). (a) FC between the left PVC and other brain regions. (b) FC between the right PVC and other brain regions. The red region indicates that the ALFF of the amblyopia group is significantly higher than that of the HCs. The blue region indicates that the ALFF of the amblyopia group is significantly lower than that of the HCs.

The FC results between the left PVC and the whole brain are shown in Figure 2(a), and the brain area statistics are provided in Table 4. In comparison with the HCs, the amblyopia group revealed that the brain regions with strong FC were mainly in the bilateral fusiform gyrus, but brain regions with a weak connectivity were mainly in the right middle frontal and angular gyri.

The results of FC between the right PVC and the whole brain are shown in Figure 2(b), and the brain area statistics are listed in Table 5. In comparison with HCs, the amblyopia group indicated that the brain regions with a strong FC were only in the right fusiform gyrus, but the brain regions with a weak connectivity were mainly in the right putamen, left orbital inferior frontal gyrus, dorsolateral superior frontal gyrus, left medial superior frontal, right angular gyrus, and right middle frontal gyrus.

The bilateral PVC had a weak connectivity with the right angular and right middle frontal gyri in amblyopia.

### 3.3. Comparison of FC between ROIs in Amblyopia versus HCs

**Figure 3:**
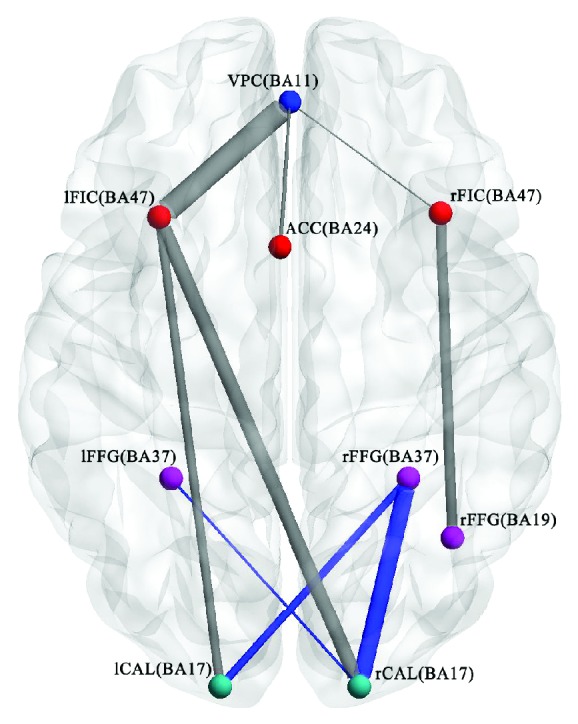
Differences in the FC of ROIs between the amblyopia group and HCs (*P* ≤ 0.05, FDR corrected, and cluster size > 85). Blue indicates that the FC strength in the amblyopia group is enhanced compared with that in HCs. Gray implies that the FC strength in the amblyopia group is weakened compared with that in HCs. VPC: ventromedial prefrontal cortex; lFIC: left frontoinsular cortex; rFIC: right frontoinsular cortex; ACC: anterior cingulate cortex; lFFG: left fusiform gyrus; rFFG: right fusiform gyrus; lCAL: left calcarine cortex; rCAL: right calcarine cortex.

Pearson's correlation was conducted for each ROI pair to assess the strength of functional coupling among network nodes.  Figure 3 illustrates the results of the two-sample *t*-test for the FC of ROIs between amblyopia and HCs.  Table 6 shows the statistical information.

In comparison with HCs, the amblyopia group possessed a weak FC between the following ROIs: left frontoinsular cortex-ventromedial prefrontal cortex, left frontoinsular cortex-left calcarine cortex, left frontoinsular cortex-right calcarine cortex, ventromedial prefrontal cortex-anterior cingulate cortex, ventromedial prefrontal cortex-right frontoinsular cortex, and right frontoinsular cortex-right fusiform gyrus. Strong FC existed between the PVCN and HVCN (left calcarine cortex-right fusiform gyrus, right calcarine cortex-left frontoinsular cortex, and right calcarine cortex-right frontoinsular cortex).

## 4. Discussion

Amblyopia shows evident visual function impairment but does not have typical ocular organic changes, although previous rs-fMRI studies have found alteration of spontaneous brain activity in amblyopia. However, fMRI studies focusing on unilateral amblyopia is limited. Here, we examined the brain functional differences between the amblyopia group and the HCs from multiple level analysis.

As a local method, ALFF analysis was conducted to locate the dysfunctional brain regions of amblyopia. In comparison with HCs, the amblyopia group indicated low ALFF in the bilateral frontal, temporal, and occipital lobes. This result was consistent with a task-related fMRI study in amblyopia [45]. The three lobes play important roles in the visual pathway. ALFF reduction in these regions may indicate a change in visual function in amblyopia. The frontal lobe plays a significant role in visual information perception, memory, and regulation [46]. The temporal lobe plays an important role in visual perception, facial recognition, and memory association and formation [47]. The occipital lobe is mainly responsible for the functions of visual and motion perception, and occipital lobe damage may cause partial or complete blindness [48, 49]. Bilateral middle temporal and middle occipital gyri are involved in the visual spatial information processing network.

The brain regions, including the fusiform gyrus, caudate nucleus, and superior parietal gyrus, with higher ALFF than HCs were on the right. Our results were different from those of Liang et al. [26], except for the left middle occipital gyrus. However, our values were reduced in the left middle occipital gyrus, whereas those of Liang et al. were increased possibly because our subjects were having unilateral left eye amblyopia, and those of Liang et al. [26] were having a mixture of unilateral left and right eye amblyopia.

We investigated whether the left/right PVC-FC with the whole brain is also altered. Our results revealed that the FC of the PVC to the right angular gyrus and the right middle frontal gyrus were significantly reduced. These regions were all in the visual pathways, possibly indicating the alteration of the PVC-FC in amblyopia. The fMRI analysis in another study [29] also showed that the FC between the PVC and angular gyrus was significantly reduced.

We used the ROIs-FC to analyze whether left eye amblyopia altered the typical brain functional networks. The results showed six reduced FC and three increased FC for amblyopia. The reduced FC was mainly located in the left hemisphere, whereas the increased FC was in the right hemisphere. A bilateral PVC has a weak connectivity with the frontoinsular cortex of the SN. The frontoinsular cortex is regarded as an information integration hub of the SN [14, 50]. The ventral stream passes through the PVC to the frontoinsular cortex. Therefore, the weak connectivity between the bilateral PVC and the frontal-insular cortex of the SN might indicate that the information integration hub of the brain was affected in amblyopia. Moreover, amblyopia might alter the FC of the DMN, SN, PVCN, and HVCN.

The results of these analyses revealed that unilateral amblyopia might alter local brain activity and FC. Amblyopia affects the dorsal and ventral stream of the visual pathway. The findings of the ALFF and PVC-FC analyses showed the reduced ALFF and FC of the angular gyrus of amblyopia. The angular gyrus is a key region of the temporoparietal junction (TPJ) [51], though no standardized anatomical definitions exist in TPJ localization [52]. As TPJ plays a critical role in the integration of top-down and bottom-up attentional controls [53], the reduced ALFF values and FC of the angular gyrus might indicate that amblyopia affected the top-down attentional control.

The PVC-FC and ROIs-FC analyses also confirmed an increased FC between the PVC and the fusiform gyrus. Similarly, the ALFF values in the right fusiform gyrus increased. These results were different from our expectations, and one possible explanation was that PVC belonged to the PVCN, whereas the fusiform gyrus belonged to the HVCN. These results might indicate that FC between the PVCN and HVCN increased. Our hypothesis was that a normal FC between the PVCN and HVCN could form a normal visual perception when visual information arrived at the PVCN for people with normal vision. However, an increased FC between the PVCN and HVCN might be necessary to form a near-normal visual perception when visual information arrived at the PVCN for the amblyopia group.

The present study has several limitations. First, the number of people enrolled in the experiment was relatively small because of the difficulty in recruiting participants and the poor controllability of the test data of children's test subjects. In the future, we can increase the sample size to reduce the possibility of making a type 1 error. Second, the current study only included left eye amblyopia, which might reduce the inferential effect on amblyopia in the right eye. In future studies, the right eye amblyopia should also be examined to compare the differences between the two groups and HCs. Thus, additional data can be provided to help understand amblyopia and neuroplasticity. These limitations are all aspects of improvement in future research.

## 5. Conclusions

Our multiple level analysis of rs-fMRI reveals that unilateral amblyopia may alter local brain activity and FC. A reduced activity in the angular gyrus may indicate that amblyopia affects the top-down attentional control. Our study may also help elucidate the neurological mechanisms of amblyopia.

## Figures and Tables

**Table 1:** Demographic information of subjects.

Subject	Gender	Age	Amblyopic type	Amblyopic eye	CVA (logMAR)		History of treatment
					OD	OS	
Amb 01	F	12	ANA	OS	-0.1	0.7	None
Amb 02	M	5	ANA	OS	0.1	0.4	None
Amb 03	F	14	ANA	OS	0.0	1.0	None
Amb 04	M	8	AMA	OS	-0.1	0.5	None
Amb 05	M	6	ANA; AMA	OS	0.0	1.0	None
Amb 06	M	13	ANA	OS	0.0	0.4	None
Amb 07	M	8	ANA; AMA	OS	0.1	0.7	None
Amb 08	M	10	ANA; AMA	OS	0.0	0.7	None
Amb 09	F	14	ANA; AMA	OS	0.0	0.5	None
Amb 10	M	12	ANA	OS	0.0	1.2	None
Amb 11	F	8	ANA	OS	0.0	0.7	None
Amb 12	M	24	AMA	OS	0.0	0.2	None
Amb 13	M	15	AMA	OS	0.0	0.5	None
Control 01	F	6	None	None	0.2	0.1	None
Control 02	F	24	None	None	0.0	0.0	None
Control 03	F	12	None	None	0.0	0.0	None
Control 04	F	12	None	None	0.0	0.0	None
Control 05	M	9	None	None	0.0	0.0	None
Control 06	F	8	None	None	0.0	0.0	None
Control 07	M	13	None	None	0.0	0.0	None
Control 08	M	14	None	None	0.0	0.0	None
Control 09	F	14	None	None	-0.2	-0.1	None
Control 10	F	11	None	None	0.0	0.0	None
Control 11	M	10	None	None	-0.1	-0.2	None
Control 12	M	7	None	None	0.0	-0.1	None
Control 13	M	10	None	None	-0.2	-0.1	None

Note: Amb: amblyopia group; F: female; M: male; CVA: corrected visual acuity; OD: oculus dexter; OS: oculus sinister; ANA: anisometropic amblyopia; AMA: ametropic amblyopia.

**Table 2:** Key nodes of each network.

Network	Region	BA	Peak MNI coordinates (mm)			*z*-scores
			*X*	*Y*	*Z*	
SN	rFIC	47	38	20	1	14.32
	lFIC	47	-33	19	-1	12.21
	ACC	24	-2.5	11.5	38.5	14.48

CEN	rDPC	9	48	22	45	15.64
	lDPC	9	-43.5	23.5	41.5	12.35
	rPPC	40	56	-50	43	14.87
	lPPC	40	-43.5	-52.5	49.5	11.41

DMN	VPC	11	0	-48	-15	14.08
	PCC	23/30	-6	-49	29	13.36

PVCN	rCAL	17	17.5	-99.5	4.5	12.10
	lCAL	17	-17.5	-99.5	4.5	13.65

HVCN	rLING	19	13	-55	-2	14.54
	rFFG	19	41	-62	-18	13.54
	rFFG	37	30	-47	-12	10.96
	lFFG	37	-30	-47	-12	12.05

Note: BA: Brodmann's area; MNI: Montreal neurological institute; SN: salience network; CEN: cerebellum network; DMN: default mode network; PVCN: primary visual cortex network; HVCN: higher visual cortex network; rFIC: right frontoinsular cortex; lFIC: left frontoinsular cortex; ACC: anterior cingulate cortex; rDPC: right dorsolateral prefrontal cortex; lDPC: left dorsolateral prefrontal cortex; rPPC: right posterior parietal cortex; lPPC: left posterior parietal cortex; VPC: ventromedial prefrontal cortex; PCC: posterior cingulate cortex; rCAL: right calcarine cortex; lCAL: left calcarine cortex; rLING: right lingual gyrus; rFFG: fusiform gyrus; lFFC: fusiform gyrus.

**Table 3:** Differences in ALFF between the amblyopia group and HCs.

Region	BA	Peak MNI coordinates (mm)			Cluster size	*T* value
		*X*	*Y*	*Z*		
rITG	20	48	-33	-18	231	-4.0713
lMTG	20	-45	-24	-18	192	-4.4553
lMFG	10	-6	57	-6	103	-4.2173
rMFG	—	39	54	-15	159	-4.2101
lMOG	19	-27	-81	9	242	-5.6561
lSFG	—	-18	63	3	149	-4.5657
lCUN	—	0	-84	42	122	-3.6131
lIPG	40	-48	-54	48	136	-3.867
lIPG	—	-24	-51	54	93	-4.5914
lPreCG	—	-24	-15	54	75	-4.3692
rPreCG	7	12	-60	63	35	-3.5482
lPCL	6	-6	-27	69	15	-2.4483
rFFG	—	33	-60	-18	222	4.881
rCAU	—	12	18	3	279	5.0006
rSPG	—	16	-63	72	322	6.5872

Note: BA: Brodmann's area; MNI: Montreal neurological institute; rITG: right inferior temporal gyrus; lMTG: left middle temporal gyrus; lMFG: left middle frontal gyrus; rMFG: right middle frontal gyrus; lMOG: left middle occipital gyrus; lSFG: left superior frontal gyrus; lCUN: left cuneus; lIPG: left inferior parietal gyrus; lPreCG: left precentral gyrus; rPreCG: right precentral gyrus; lPCL: left paracentral lobule; rFFG: right fusiform gyrus; rCAU: right caudate nucleus; rSPG: right superior parietal gyrus.

**Table 4:** Regions with statistically significant FC to the left PVC between the amblyopia group and HCs.

Region	BA	Peak MNI coordinates (mm)			Cluster size	*T* value
		*X*	*Y*	*Z*		
rFFG	37	36	-48	-12	72	5.9695
lFFG	37	-33	-51	-6	85	5.1071
rMFG	—	39	39	33	82	-4.1831
rANG	—	42	-69	54	72	-4.3852

Note: BA: Brodmann's area; MNI: Montreal neurological institute; rFFG: right fusiform gyrus; lFFG: left fusiform gyrus; rMFG: right middle frontal gyrus; rANG: right angular gyrus.

**Table 5:** Regions with statistically significant FC to the right PVC between the amblyopia group and HCs.

Region (aal)	BA	Peak MNI coordinates (mm)			Cluster size	*T* value
		*X*	*Y*	*Z*		
rFFG	37	36	-48	-9	82	6.6431
rPUT	—	18	9	-6	178	-3.8243
lORBinf	—	-39	42	-6	91	-3.8903
rSFG	—	21	57	3	353	-4.718
lSFGmed	—	-9	30	51	130	-6.1944
rANG	40	42	-69	54	95	-4.1872
rMFG	8	21	27	42	215	-5.6782

Note: BA: Brodmann's area; MNI: Montreal neurological institute; rFFG: right fusiform gyrus; rPUT: right lenticular nucleus-putamen; lORBinf: left orbital part inferior frontal gyrus; rSFG: right superior frontal gyrus; lSFGmed: right medial superior frontal gyrus; rANG: right angular gyrus; rMFG: right middle frontal gyrus.

**Table 6:** Difference in FC between ROIs in important networks of amblyopia.

	VPC (BA11)	lFIC (BA47)	rFIC (BA47)	ACC (BA24)	lFFG (BA37)	rFFG (BA37)	lCAL (BA17)	rCAL (BA17)	rFFG (BA19)
VPC (BA11)		↓	↓	↓					
lFIC (BA47)	↓						↓	↓	
rFIC (BA47)	↓								↓
ACC (BA24)	↓								
lFFG (BA37)								↑	
rFFG (BA37)							↑	↑	
lCAL (BA17)		↓				↑			
rCAL (BA17)		↓			↑	↑			
rFFG (BA19)			↓						

Note: “↓” indicates a decrease in FC in the amblyopia relative to the HCs. “↑” denotes an increase in FC in the amblyopia relative to the HCs. VPC: ventromedial prefrontal cortex; lFIC: left frontoinsular cortex; rFIC: right frontoinsular cortex; ACC: anterior cingulate cortex; lFFG: left fusiform gyrus; rFFG: right fusiform gyrus; lCAL: left calcarine cortex; rCAL: right calcarine cortex.

## Data Availability

The resting state fMRI data used to support the findings of this study were supplied by the Ethics Committee of the Second Xiangya Hospital under license and so cannot be made freely available. Requests for access to these data should be made to Professor Xiao (13973119862@163.com).
